# The role of local protein synthesis and degradation in axon regeneration

**DOI:** 10.1016/j.expneurol.2009.06.004

**Published:** 2010-05

**Authors:** Laura F. Gumy, Chin Lik Tan, James W. Fawcett

**Affiliations:** Cambridge Centre for Brain Repair, Department of Clinical Neuroscience, University of Cambridge, UK

**Keywords:** Axon regeneration, Local protein synthesis, Local degradation, Growth cone formation

## Abstract

In axotomised regenerating axons, the first step toward successful regeneration is the formation of a growth cone. This requires a variety of dynamic morphological and biochemical changes in the axon, including the appearance of many new cytoskeletal, cell surface and signalling molecules. These changes suggest the activation of coordinated complex cellular processes. A recent development has been the demonstration that the regenerative ability of some axons depends on their capacity to locally synthesise new proteins and degrade others at the injury site autonomously from the cell body. There are also events involving the degradation of cytoskeletal and other molecules, and activation of signalling pathways, with axotomy-induced calcium changes probably being an initiating event. A future challenge will be to understand how this complex network of processes interacts in order to find therapeutic ways of promoting the regeneration of CNS axons.

## Introduction

Injury to the nerve results in disconnection of the cell body from the distal target. In such injuries, axons are broken into at least two major fragments. The distal portion becomes physically separated from the cell body and undergoes Wallerian degeneration, culminating in its degradation. The proximal portion, which is still attached to the cell body, usually initiates a regenerative phase in order to re-grow towards its targets. This phenomenon of axon regeneration is much more successful in the peripheral nervous system (PNS), as compared to the central nervous system (CNS). In the former, damaged axons are generally capable of re-growing a long distance to result in functional recovery. On the contrary, neurons from the CNS are notoriously poor at regenerating axons after an injury. Regeneration requires the re-creation of the axon growth cone, a specialised motile structure that contains many molecules and signalling pathways that are not found in the mid part of mature axons. Much effort has been put into understanding the processes and mechanisms that underlie axon regeneration particularly in the PNS, with the hope of translating some useful knowledge into improving regeneration of the CNS. In this review, we aim to provide a summary of the current understanding of the roles of one of the mechanisms, i.e. local axonal protein synthesis and protein degradation in mediating axon regeneration.

### Axon dynamics after injury

As a result of transection, the plasma membrane of the cut axon tip becomes damaged, and the axoplasm is exposed to the extracellular environment. This causes leakage of intracellular contents or the influx of materials in the opposite direction, either of which may be detrimental to the neuron. Therefore, to achieve successful regeneration, the axon first needs to re-seal the damaged membrane of the proximal tip to preserve the intra-axonal milieu (Eddleman et al., 2000; Spira et al., 1993, 1996). Then, the neuron must be able to convert the axon into a new growth cone. This is achieved by a highly dynamic process involving the remodelling of the intracellular cytoskeleton inside the axon, as well as the modification of the membrane and membrane-associated molecules, to transform it from a cylindrical tube into an expansive, dynamic, and motile structure that can grow through the surrounding environment and respond to growth and guidance cues (Sahly et al., 2006).

Events leading up to the formation of a growth cone post-axotomy have been studied in greatest depth in cultured *Aplysia* neurons. First, the proximal stump degenerates a small extent as the membrane seals, associated with the activation of calpains and degradation of cytoskeletal elements. This is followed by the appearance of an area of swelling or enlargement near the axon terminal within 5 min (Spira et al., 2003; Verma et al., 2005). In the invertebrate *Aplysia*, the axon then becomes broadly divided into 3 zones—namely proximal, transition and distal zones—correlating with the distance from the cell body. This happens as a result of a reorganisation of the cytoskeleton via the breakdown and re-polymerisation of microtubules, neurofilaments and actin filaments (Spira et al., 2003). In the proximal zone, the cytoskeletal structure remains intact; whereas in the distal zone, the breakdown products of microtubules and neurofilaments form electron-dense aggregates (Sahly et al., 2006; Spira et al., 2003). The transition zone undergoes an initial accumulation of vesicles due to the disruption to the transport system, and later becomes the organising centre for the nascent growth cone (Spira et al., 2003). Then, actin filaments polymerise along the periphery of the transition zone, forming the expanding lamellipodium of the growth cone (Sahly et al., 2006; Spira et al., 2003). Further polymerisation of cytoskeletal elements is believed to support the re-structuring of the growth cone and the regeneration of the axon.

The events following axotomy of mammalian neurons *in vitro* are probably similar, but have not been described in the same degree of detail as for *Aplysia*. There is an initial axonal withdrawal from the site of axotomy, usually followed in sensory neurons by the regeneration of a new growth cone (Chierzi et al., 2005; Verma et al., 2005). When comparing neurons cultured from rat dorsal root ganglion (DRG) neurons and retinal ganglion cells (RGCs), it was found that the terminal swelling in DRG axons are mostly transformed into a new growth cone, but those from RGCs tend not to do so (Verma et al., 2005). This is consistent with the widely-held idea that CNS (i.e. RGC) neurons have a lower regenerative capacity compared to PNS (i.e. DRG) neurons. Besides, there is also a progressive developmental decline of regenerative ability, reflecting the effect of ageing on regeneration (Chierzi et al., 2005; Verma et al., 2005).

The observation that transected axons undergo a series of processes involving retraction, terminal enlargement formation, growth cone re-formation and axon extension, indicates that a dynamic protein turnover event must be occurring. New proteins are being synthesised while old ones are degraded. This interplay between protein synthesis and degradation must be tightly regulated so as to orchestrate the transformation of the axonal terminal stump into a well-formed growth cone in an orderly fashion.

## The role of protein synthesis in axon regeneration

### The identification and localisation of protein synthesis machinery in axons

Recent work from our laboratory attempted to find out the role, if any, of local axonal protein synthesis during axon regeneration after axotomy. Using DRG neuron cultures, we severed the axons extending from the explants with a glass electrode and observed with time-lapse microscopy (Verma et al., 2005). We saw that many neurons successfully re-form new growth cones within just 20 min post-axotomy, and when analysed after 4 h, a large percentage of DRG neurons (∼ 80% in embryos, ∼ 70% in adults) had successfully regenerated a new growth cone (Verma et al., 2005). Such a fast response argues for a local regulation of regenerative events post-axotomy, because most molecules are not transported fast enough for newly synthesised material to have arrived from the cell body. Indeed, virtually identical results were obtained when the growth cone regeneration assay was carried out on axons that had been disconnected from their cell bodies, thus ruling out any overriding control from there. Importantly, the application of protein synthesis inhibitors, cycloheximide and anisomycin, significantly impairs the growth cone regenerative ability (Verma et al., 2005). This implies that the ability of axons to undertake local protein synthesis could underlie their capability to regenerate post-axotomy, at least in the early stages (within 4 h). And to achieve that, it means that machinery that carries out protein synthesis must be in place beforehand, or must be rapidly localised to the injured site.

Evidence of the axonal localisation of protein synthesis machinery came from several studies. Early reports demonstrated the localisation of mRNAs, tRNAs, rRNAs and elongation factors to the squid giant axon and the Mauthner axon of the goldfish. Later, regenerating adult rat sensory axons were shown to contain several ribosomal proteins (P0, L4, L29, L17 and RPP) in addition to translation initiation factors (eIF2α, eIF4e and eIF5) (reviewed by Piper and Holt, 2004; Verma et al., 2005; Zheng et al., 2001). Although initial attempts to detect ribosomes in mature axons using conventional electron microscopy failed, with the development of high-resolution electron microscopy techniques, the presence of ribosomes was observed in the axoplasmic compartment of invertebrate and mammalian vertebrate axons. The ribosomes appeared to be organised into plaque-like structures on the outer boundary of the axoplasm and colocalised with a cytoskeletal matrix composed of F-actin (Koenig and Martin, 1996; Koenig et al., 2000; Kun et al., 2007; Pannese and Ledda, 1991; Sotelo et al., 1999). In addition, immunostaining with the Y10B antibody against 28 s rRNA detected the presence of rRNA in the axons (Lerner et al., 1981; Zheng et al., 2001). Recently, convincing evidence of the localisation of ribosomes to adult axons comes from the work of Court et al. (2008). In this study, endogenous ribosomes (presumably transported from the cell body) were detected in the mouse sciatic nerve. Surprisingly, after nerve damage Schwann cells appeared to transfer their own ribosomes into the axon.

**Fig. 1:**
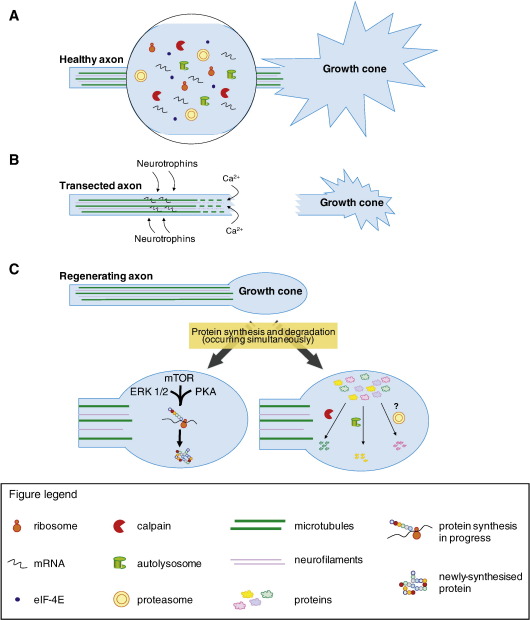
(A) Inside a healthy axon, a variety of protein synthesis and degradation machinery is present. (B) As a result of transection, the axon is divided into two parts. The distal portion undergoes Wallerian degeneration and will subsequently be degraded. In the proximal portion, calcium enters the axon due to the disruption to the plasma membrane, as well as via voltage-gated calcium channels. Cytoskeletal structures such as microtubules and neurofilament undergo depolymerisation and degradation. Neurotrophins from extracellular sources may also help the localisation of mRNAs. (C) Later on, a terminal swelling appears at the tip of the proximal stump, as regeneration ensues. Microtubules and neurofilaments undergo re-polymerisation. Protein synthesis and protein degradation occur simultaneously within the axon. Protein synthesis takes place via a mechanism dependent on mTOR, ERK 1/2 and PKA. Examples of proteins synthesised locally after an injury include importin b1, vimentin, ribosomal protein L4 etc. Protein degradation may occur via calpain-dependent proteolysis (e.g. vimentin, spectrin) or ubiquitin–proteasome system, while the role of autophagy remains to be elucidated.

To date several hundred mRNAs have been identified and localised to axons (Fig. 1A). Initially, the identification was carried out in invertebrate axons (Chun et al., 1995, 1997; Gioio et al., 2004; Giuditta et al., 1991; reviewed in Alvarez et al., 2000). Currently, approximately 40 different mRNAs have been detected, including mRNAs for ribosomal proteins, cytoskeletal proteins, motor proteins, translation factors, nuclear-encoded mitochondrial proteins and many others (Gioio et al., 2004). In mammalian axons, a large number of mRNAs have been identified by the combined use of molecular biology, metabolic labelling and new proteomic methodologies (Eng et al., 1999; Willis et al., 2005, 2007). Preliminary studies focused on developing axons (Bassell et al., 1998; Cox et al., 2008; Eng et al., 1999; Olink-Coux and Hollenbeck, 1996; Wu et al., 2005) and later on regenerating adult sensory and motor axons *in vitro* and *in vivo* (Hanz et al., 2003; Koenig et al., 2000; Perlson et al., 2005; Verma et al., 2005; Willis et al., 2005, 2007; Yudin et al., 2008; Zheng et al., 2001). By RT-PCR the mRNAs of cytoskeletal proteins (such as β-actin, β-tubulin, vimentin, NF-L), heat shock proteins, resident endoplasmic reticulum (ER) proteins and other mRNAs encoding proteins related to neurodegenerative diseases and metabolism were detected (Willis et al., 2005; Zheng et al., 2001).

To increase the number of mRNAs identified in axons, Willis et al. (2007) extracted mRNAs from pure axonal preparations and reverse transcribed these into cDNAs that were subsequently amplified. These were then hybridised onto cDNA arrays that contain about 4000 rat cDNAs. Their study revealed that over 200 mRNAs were present in the adult sensory axons of injury-conditioned DRG. We (L. Gumy and J.W. Fawcett; unpublished data) have recently characterised mRNAs from uninjured adult sensory axons and functionally classified the mRNAs by gene ontology. Remarkably, the most abundant functional groups appeared to be the ribosomal/translational machinery and the mitochondrial/oxidative phosphorylation ones. About 58 mRNAs for ribosomal proteins were present in the array out of 80 rat ribosomal proteins that have been described to date (Wool et al., 1995). Interestingly, several components of the ubiquitin–proteasome system appeared clustered together, including ubiquitin c. Curiously, some nuclear proteins such as histones and transcription factors were also present and several signalling proteins and cytoskeletal components were detected, corroborating previous reports. Recently, Taylor et al. (2009) provided evidence that CNS axons also contain many localised mRNAs of diverse function. Interestingly, the authors found that the composition of axonal mRNAs changed after axonal injury and during regeneration, where an increase in transcripts related to axonal outgrowth, targeting and synapse formation was observed (for more details on injury-induced RNA transport see review by Yoo et al., 2010). Many of the mRNAs found in naive and injured CNS axons match those reported in PNS axons. For example, CNS axons also appear to contain a high number of transcripts related to ribosomal proteins as reported in previous studies. Similarly, in invertebrate axons it also appears that some of the most abundant and highly represented mRNAs encode for ribosomal proteins (Gioio et al., 2004). Perhaps future work will highlight whether mRNAs encoding ribosomal proteins are locally translated and if so whether ribosomal proteins can exert functions specifically adapted to the physiology of the axon and growth cone.

The possible mechanisms for axonal mRNA sorting and transport have been extensively reviewed (Bassell and Kelic, 2004; Du et al., 2007; Kindler et al., 2005; Sossin and DesGroseillers, 2006; Sotelo-Silveira et al., 2006; Wang et al., 2007). Briefly, active sorting and transport involves the identification of specific sequences and structures within the mRNA (e.g. “zipcode” in the 3′ untranslated region (UTR) of β-actin) by RNA-binding factors. The interaction between the mRNA and the RNA-binding factors (e.g. Staufen, kinesins) results in the formation of ribonucleotide complexes that travel along axonal microtubules. The specific targeting of the mRNA (e.g. dendrites or axons in CNS neurons) appears to depend on the targeting elements within the mRNA, the RNA-binding factors present and the activity of the neuron. Below, we examine recent findings on the localisation of mRNAs by extracellular cues during regeneration.

Willis et al. (2005) showed that the bath application of neurotrophins increased the transport of β-actin mRNA to the growth cone. Subsequently, it was shown that several neurotrophins (NGF, BDNF, NT-3) as well as inhibitory molecules (MAG, Sema3A) modified the levels of particular mRNAs in the axons (Willis et al., 2007; Fig. 1B). Very elegantly it was reported on how these ligands specifically increased or decreased the localisation of 50 mRNAs that belong to a wide range of protein families. Furthermore, it was found that the specific ligand-induced localisation of the mRNAs in the axon was independent of new transcription and correlated with mRNA changes in the cell body. Consequently, it was established that extracellular stimuli could target and redistribute pre-existing mRNAs through the Trk-PI3K-MEK1 signalling pathways to specific locations in the axon. The movement of mRNAs appeared to depend on microtubule and microfilament transport (Willis et al., 2007). These findings set a new scenario on axonal regeneration. It is now evident that extracellular stimuli (growth-promoting and inhibitory) can differentially localise specific mRNAs in the axons. This could allow the possibility of manipulating the localisation of mRNAs to promote regeneration such as in the CNS where many axons are intrinsically incapable of regenerating even in a permissive environment.

The modulation of gene expression by the microRNA-mediated silencing machinery is gaining functional importance in neurons (Kosik, 2006). Indeed, recent results show that proteins involved in the processing of miRNAs are localised to developing DRG axons and growth cones and to mature peripheral axons *in vivo*. The microRNA silencing machinery was found to be functional in axons and mediated gene silencing of specific mRNAs (Hengst et al., 2006; Murashov et al., 2007). This raised the possibility that axonal microRNAs may be a feature of mRNA translational regulation and hence, provide alternative means for regulating axon regeneration (Hengst et al., 2006). Interestingly, the microRNA miR132 was shown to be enriched in neurons and its expression was upregulated by neurotrophins (Vo et al., 2005). In cortical neurons miR132 expression promoted neurite outgrowth while its inhibition caused a decrease, providing evidence that microRNAs are capable of regulating neurite length. Another study (Aschrafi et al., 2008) found that miR338, a brain specific microRNA, decreased COXIV mRNA and protein levels with a concomitant decrease in mitochondrial activity in SCG neurons. The post-transcriptional regulation of gene expression by miRNAs is notable. Prospective work will establish whether a correlation can be made between axonal microRNA content/activity and axon regeneration ability.

What is lacking at present is an explanation of the apparent difference between PNS and CNS axons in local translation abilities. Immunostaining the spinal cord for ribosomal protein P0 shows that it is only present in the central branches of DRG axons, not in other CNS axons (Verma and Fawcett; unpublished results). It is possible; therefore, that many other items of translational machinery and mRNAs have a different distribution in mature CNS axons to the PNS. Finding out how to regulate the localisation of mRNAs and other translational molecules in CNS axons will certainly provide ways to promote and enhance regeneration.

### Evidence of local protein synthesis in regenerating axons

The earliest experiments suggesting that local protein synthesis occurs in regenerating axons go back to the 1960s. At that time, Koenig found that the level of acetylcholinesterase in cat hypoglossal nerve increased five-fold within 24 h after transection (neurotomy), and that this increase could be greatly reduced by local treatment of a protein synthesis inhibitor, puromycin (Koenig, 1965, 1967). This provided the first indication that the axons may possess the ability to synthesise proteins locally. Later on, with his colleagues, he verified and further characterised axonal protein synthesis activity using rabbit hypoglossal nerve transection model. They found that in the nerve region just proximal to the transection, both the protein content and protein synthesising activity (as assessed by radiolabelled leucine incorporation) were elevated after neurotomy (Tobias and Koenig, 1975a). The protein content increases gradually following nerve transection, peaks at 21 h with a doubling of protein content, and returns to initial level after 96 h. Meanwhile, the protein synthesising activity reaches a 20-fold increase at about 18 h post-neurotomy, decreases thereafter but stays at least two-fold higher compared to the initial level (Tobias and Koenig, 1975a). Interestingly, addition of the protein synthesis inhibitor cycloheximide, but not chloramphenicol (which inhibits mitochondrial protein synthesis), abolishes the protein synthesis, indicating that extra-mitochondrial protein synthesis might be at work (Tobias and Koenig, 1975a). They further showed that this protein synthesis is localised in the axon by demonstrating that ‘de-centralising’ axons—to disconnect the nerve from the cell body—has no effect on the protein synthesising activity (Tobias and Koenig, 1975b). Later on, Gaete et al. also obtained a similar result using rat peroneal nerve injury model (Gaete et al., 1998). Both groups attempted to demonstrate that the protein synthesis was axonal, by using axon samples with their myelin sheaths removed (Tobias and Koenig, 1975a), or by growing axons in an acellular environment (Gaete et al., 1998). However, the purity of axonal samples obtained via such methods could not be verified with certainty.

A similar picture has also been found to be true in *in vitro* neuronal cultures. For instance, regenerating goldfish RGCs in culture (grown in the presence of 5-fluorodeoxyuridine to remove proliferating non-neuronal cells) actively incorporate tritiated amino acids and [^35^S]methionine into axonal proteins, in a process that is inhibited by cycloheximide (Koenig, 1989; Koenig and Adams, 1982). Regeneration of a crushed adult mouse sciatic nerve *in vitro* was also shown to be partially dependent on local protein synthesis in the injury area (Edbladh et al., 1994). More recently, using two different preparations of adult rat DRG neuron cultures, a similar picture was seen. In one, axons from cultures of conditioned DRG preparations were collected and incubated in a medium containing [^35^S]methionine/cysteine. Radiolabelled proteins were consistently detected in these samples, indicating that protein synthesis was taking place (Zheng et al., 2001). In the other, after DRG axons were severed with a glass electrode, the incorporation of [^3^H]leucine into axons increased dramatically (Verma et al., 2005). Furthermore, rapamycin, another protein synthesis inhibitor, abolished this activity (see below; Verma et al., 2005).

These, along with our aforementioned observation that axotomised axons—even those isolated from their cell bodies—regenerate a new growth cone fairly rapidly, provide evidence that local axonal protein synthesis indeed occurs after injury (Verma et al., 2005).

But what are the proteins synthesised in the axons during regeneration? While the answer remains ambiguous, cytoskeletal proteins seem to be the most likely candidates. Frankel and Koenig discovered a ∼ 41 kDa protein that was synthesised in transected, but not untransected, hypoglossal nerves, and speculated that it could be actin (Frankel and Koenig, 1978). Besides, analysis of lysates from regenerating axons of conditioned goldfish RGCs suggests the local synthesis of actin and tubulin (Koenig, 1989). Studies on regenerating PC12 cells and conditioned DRGs, meanwhile, showed that the translation of ribosomal protein L4 mRNA is required for efficient axon regeneration, as knocking down the mRNAs with antisense oligonucleotides resulted in failure of regeneration (Twiss et al., 2000).

The axonal synthesis of another protein, importin β1—which belongs to the karyopherin family that regulates the translocation of nuclear localisation signal (NLS)-bearing proteins into the nucleus—has also been found to be upregulated following a nerve lesion (Hanz et al., 2003). Likewise, an intermediate filament, vimentin, is also actively synthesised after a nerve injury (Perlson et al., 2005; Willis et al., 2005). This newly–synthesised vimentin first undergoes proteolysis, and the proteolytic fragments in turn link up with importin β1, and together they are retrogradely transported to the cell body (Perlson et al., 2005). While being transported, they help to ferry a cargo, now identified as phosphorylated ERK 1/2 (pErk), which may have a signalling function in the cell body (Perlson et al., 2005; Reynolds et al., 2001; Sung et al., 2001). It is therefore noteworthy that rapid phosphorylation of ERK 1/2 takes place after an injury (Chierzi et al., 2005; see below). (For a more detailed account on this theme, see review by Rishal and Fainzilber, 2010.)

Recently, Willis et al. attempted to catalogue the axonally-synthesised proteins in DRG neurons cultured from adult rats that had been injury-conditioned by a sciatic nerve crush (also see review by Yoo et al., 2010). By employing a proteomics approach to analyse axonal lysates from the injury-conditioned animals, more than 100 proteins were identified (Willis et al., 2005). As expected, a number of cytoskeletal proteins were among them, including β-actin, peripherin, vimentin, γ-tropomyosin 3 and cofilin-1 (Willis et al., 2005). Surprisingly, many non-structural proteins were also found to be synthesised in the axons. These include heat shock proteins, resident ER proteins, anti-oxidant proteins and metabolic proteins (Willis et al., 2005). Working on cultures of condition-injured DRG neurons of *Xenopus laevis*, Tonge et al. metabolically labelled the axons in a compartmentalised chamber, and subsequently collected the cell bodies for analysis (Tonge et al., 2008). Using mass spectrometry, they identified 35 proteins that were synthesised in the axons and transported retrogradely back to the cell bodies, including cytoskeletal/cytoskeletal-binding proteins, calcium ion-binding proteins, annexins, translation factors, and the ubiquitin–proteasome system (Tonge et al., 2008). The pool of newly synthesised cytoskeletal proteins is likely to act as a source of structural proteins for growth cone re-formation and axon growth (Willis and Twiss, 2006; Zhang et al., 2001), as well as to regulate cytoskeletal dynamics (Bamburg et al., 1999; Ghosh et al., 2004). Further studies are necessary to discover the roles, if any, of these proteins in the context of nerve injury and axon regeneration.

Attempts were also made to understand the mechanism that mediates the local protein synthesis (Fig. 1C). In view of the involvement of mammalian target of rapamycin (mTOR) and the mitogen-activated protein kinase p38 in regulating local protein synthesis in axon guidance, Verma et al. (2005) investigated if a similar mechanism is employed in axon regeneration. In cultures of DRG neurons and RGCs, addition of the inhibitors to these molecules significantly diminishes both the incorporation of [^3^H]leucine into axonal proteins, and the regeneration of new growth cones after axotomy (Verma et al., 2005). Recently, He and co-workers created mice in which PTEN (phosphatase and tensin homolog), a negative regulator of mTOR pathway, was removed from the RGCs via a conditional knockout approach. They found that, when compared to wild-type mice, these animals exhibited better regeneration of RGC axons after an optic nerve injury (Park et al., 2008). Although in this case, the effect of the elimination of PTEN on mRNA translation is a global one, the localised protein synthesis within the axon would also be affected, thus not necessarily negating a role for local protein synthesis in axon regeneration.

Other signalling molecules such as extracellular signal-regulated kinase 1,2 (ERK 1/2) and protein kinase A (PKA) were also found to be important in ensuring successful growth cone regeneration of DRG neurons axotomised *in vitro* (Chierzi et al., 2005). Although the association with protein synthesis is not immediately clear, it was speculated that they may be involved in the synthesis of growth-related proteins from axonally-localised mRNAs, not unlike the role of ERK in regulating growth cone chemotropic responses to diffusible cues (Campbell and Holt, 2003; Chierzi et al., 2005). Alternatively, activated forms of signalling molecules (e.g. pErk) can be retrogradely transported to the cell body, and may act as an ‘injury signal’, informing the cell body of the injury occurring distally (Perlson et al., 2005; Reynolds et al., 2001; Sung et al., 2001). Besides, calcium also appears to be crucial for successful axon regeneration (Chierzi et al., 2005; Yudin et al., 2008; see below). It was shown that calcium is required to regulate the binding of the components of the importin retrograde signalling complex; and may also be involved in the translation of these components such as importin β1 and vimentin (Perlson et al., 2006; Yudin et al., 2008).

To determine if there is any relationship between the regenerative capacity of different neurons of varying ages and local protein synthesis, the levels of various protein synthesis machineries within the axons had been compared. By performing quantitative immunofluorescence (QIF) assays on cultured DRG neurons and RGCs, it was found that the levels of ribosomal protein P0 and phosphorylated initiation factor eIF-4E in the axons decrease with developmental age. Moreover, the levels in the CNS (i.e. RGCs) are much lower compared to those in the PNS (i.e. DRG neurons) (Verma et al., 2005). This could well explain the progressive loss of regenerative potential as neurons age as well as the limited ability of CNS neurons to regenerate as compared to PNS neurons. Indeed, in adult mouse RGCs, it was observed that the mTOR activity and new protein synthesis was impaired after an axotomy, which may result in the failure of regeneration. Nevertheless, in mice in which tuberous sclerosis complex 1 (another negative regulator of the mTOR pathway) was conditionally knocked out, robust regeneration ensued (Park et al., 2008). This suggests that promoting protein synthesis could be an effective approach to enhance regenerative capacity, even in neurons that normally lack such ability.

## The role of protein degradation in regenerating axons

Axotomised regenerating axons undergo rapid dramatic anatomical changes *in vitro* and *in vivo*. As previously mentioned, soon after transection, the axons retract, re-form a growth cone and elongate forward. These morphological and functional changes are accompanied by a rapid turnover of proteins involving a dynamic balance between local synthesis of new proteins and degradation of pre-existing ones. In the past decade, local protein synthesis in the axon has increasingly gained attention; however, the role of protein degradation in axon regeneration has been given less consideration.

### Role of the ubiquitin–proteasome system in axon regeneration

The degradation of proteins in the axon can take place by different proteolytic pathways that include the ubiquitin–proteasome system (UPS), calcium-mediated proteolysis and autophagy-mediated degradation. Of these proteolytic systems, the UPS has been described as a major player in regulating a multitude of cell processes and dynamics; increasing evidence has also shown that its impairment may lead to neuronal dysfunction (extensively reviewed by Glickman and Ciechanover, 2002; Johnston and Madura, 2004; Murphey and Godenschwege, 2002; van Tijn et al., 2008).

Briefly, the UPS comprises several essential components. The first one is ubiquitin, a 76 amino acid polypeptide. Through a series of complex multi-step reactions, ubiquitin is activated by the E1 ubiquitin-activating enzyme and transferred to the ubiquitin carrier, the E2 ubiquitin conjugating enzyme. The E3 ubiquitin ligase enzymes will recognise proteins to be targeted for degradation by the proteasome and will either transfer the ubiquitin directly to a lysine residue at position 48 on the protein substrate or transfer ubiquitin from the E2 enzyme to the E3 ligase and then to the substrate. Protein substrates tagged with multi-ubiquitin chains are then selectively degraded by the 26S proteasome into small peptides. The 26S proteasome is a multi-subunit protease complex, composed of a 20S core particle and by at least one 19S regulatory particle. Once the target protein has been proteolytically degraded, the ubiquitin hydrolase-catalysed depolymerisation of the polyubiquitin chain will release recyclable monomeric ubiquitins. The protein substrates of the UPS are generally short-lived cytoplasmic and plasma membrane, misfolded or damaged proteins which must be structurally different from stable and essential proteins.

In the 1980s a number of studies reported axonal transport and localisation of tRNA in vertebrate axons. Interestingly, the amount of transported tRNA appeared to increase during development and regeneration (Chakraborty and Ingoglia, 1993). Although initially it was thought that tRNA contributed only to protein synthesis, later it was established that tRNA also worked as an amino acid donor that posttranslationally argynilates proteins at the N-terminus for ubiquitin-mediated proteolytic degradation (Chakraborty and Ingoglia, 1993; Ferber and Ciechanover, 1987; Jack et al., 1992). In regenerating sciatic nerves, an increase in N-terminal argynilated and ubiquitinated proteins was observed (Jack et al., 1992). Conversely, in the optic nerve (poor regenerative ability), N-argynilation did not occur until several days after the injury (Shyne-Athwal et al., 1988; Shyne-Athwal et al., 1986; Zanakis et al., 1984). Thereby, it was hypothesised that the UPS might be important for nerve regeneration suggesting that UPS-mediated protein degradation occurs in regenerating axons but fails to occur in non-regenerating ones.

The elucidation of the localisation and function of the UPS machinery in the growth cones came from Campbell and Holt (2001) (Fig. 1A). In this work several components of the proteasome and its related signalling molecules (for example, 20S proteasome “core”, csn8 protein of the COP9 signalling complex (regulator of UPS) and ubiquitin) were detected by immunohistochemistry in developing RGC growth cones of *Xenopus laevis* in culture. Further studies showed that components of the UPS were present in mammalian axons, these included the ubiquitin–conjugating enzyme HR6B (Kavakebi et al., 2005), 20S proteasome “core”, ubiquitin and ubiquitinated protein (Verma et al., 2005), the Ring-ubiquitin-ligase Rnf6 (Tursun et al., 2005) and the E3-ubiquitin ligase (Lewcock et al., 2007). Apart from its presence in the axons and growth cones the UPS components were also functional. For example, treatment of isolated growth cones with proteasome inhibitors prevented chemotropic-mediated growth cone turning (Campbell and Holt, 2001). In cultured mammalian sensory and superior cervical ganglion (SCG) neurons, treatment with a variety of proteasome inhibitors (namely lactacystin, and the dipeptide Leu-Ala) reduced or inhibited axonal outgrowth of newly plated neurons while in established cultures they induced a dose-dependent decrease in axonal length (Kavakebi et al., 2005; Klimaschewski et al., 2006; Laser et al., 2003). Additionally, it was observed that proteasomal inhibition induced the fragmentation and dispersion of high molecular weight neurofilaments in the growth cones and axons of a subpopulation of neurons (Klimaschewski et al., 2006).

It was not until recently that the role of UPS-mediated degradation was assessed in growth cone formation after axotomy (Verma et al., 2005). Indeed it was shown that proteasomal inhibitors decreased the regeneration of isolated growth cones in cultured rat sensory axons throughout all developmental stages (embryonic, neonate and adult) and to a much lesser extent in adult retinal axons, which already presented limited regeneration. *In vivo* it was shown that a lesion could increase the levels of protein degradation machinery proximal to the site of injury. Thus, the UPS appeared to be necessary for growth cone regeneration (Verma et al., 2005).

Very few mechanisms have been proposed to explain how the UPS mediates axon regeneration. Nevertheless, it is widely accepted that ubiquitination events are required for correct neuronal function (Murphey and Godenschwege, 2002; van Horck et al., 2004). Attempts to explore the links between components of the UPS, signalling pathways and axonal growth cone dynamics suggest that degradation of cytoskeletal components could be a limiting step in regulating axonal regeneration (Lewcock et al., 2007; Tursun et al., 2005). For example, the expression levels of the Ring-ubiquitin-ligase Rnf6 indirectly modulate neurite length by targeting LIMK1 (a regulator of the cellular actin cytoskeleton) for proteasomal degradation by polyubiquitination (Tursun et al., 2005). A similar picture has also been found with the E3-ubiquitin ligase Phr1. Phr1 is associated with stable microtubules in the axon shaft of sensory and motor neurons. Interestingly, Phr1 regulates the levels of the DLK protein by targeting it for proteasomal degradation with consequences for the stability of microtubules and hence growth cone and axon morphology (Lewcock et al., 2007).

Although several reports have established links between the UPS and the growth cone/axon dynamics, several other questions remain open and require further exploration. For example, it appears that the conjugation of ubiquitin to a substrate is a very specific reaction that derives from specific combinations of ubiquitin conjugating (E2) and ubiquitin ligase (E3) enzymes (Watts et al., 2003) however, it is unknown how axotomy or axonal injury triggers the ubiquitination of specific proteins. Also, it is unknown how UPS-mediated protein degradation in the axon or growth cone is spatially regulated and the timeframe over which occurs after injury. For example, the vicinity of the ubiquitin ligases to the substrate or the spatial restriction of the different components of the proteasome into subcellular compartments might regulate the degradation process. Furthermore, the temporal specificity of ubiquitination will depend on the stability/instability of proteins, the posttranslational modifications such as phosphorylation and on the susceptibility of ligases to second messengers such as Ca^2+^ (see review by Hedge, 2004). For example, a transient intracellular influx of Ca^2+^ after axotomy might regulate the ubiquitination of specific proteins, in particular since a similar modulation of ubiquitination by Ca^2+^ has been described for molecules related to synaptic plasticity (Abrams et al., 1998; Onyike et al., 1998; Speese et al., 2003; Zhao et al., 2003). Recently, Staal et al. investigated the role of the UPS in mammalian CNS axons after a stretch injury (Staal et al., 2009). It was observed that after injury the accumulation of ubiquitin in these axons was temporally and spatially associated with cytoskeletal rearrangements suggesting that UPS activity might help to reorganise the cytoskeleton in CNS axons after injury.

In summary, components of the UPS are present in regenerating and non-regenerating axons. In regenerating axons it appears that UPS activity and its inhibition correlate with growth cone regeneration success and with axonal protection after injury (Verma et al., 2005; Staal et al., 2009). However, further work will need to highlight how specific substrate ubiquitination occurs after axotomy and how these events are spatially and temporally controlled since untimely and excessive degradation could become detrimental for the regeneration of the axon.

### Calcium/calpain-mediated proteolysis in growth cone re-formation

Apart from the proteasome-dependent protein degradation, extensive studies in *Aplysia* have shown that calcium/calpain-mediated proteolysis also has a major role in growth cone re-formation and axon regeneration after injury (reviewed by Spira et al., 2001). The involvement of calcium ions in the formation of growth cones has been supported by several experiments. Firstly, after an axotomy, intracellular calcium concentration, [Ca^2+^]_i_, rises rapidly at the site of transection, with the level reaching more than 1 mM (Fig. 1B). This increase in [Ca^2+^]_i_ can be attributed to the sudden influx of calcium ions into the axoplasm through the ruptured membrane (Ziv and Spira, 1995; Ziv and Spira, 1997), the opening of voltage-gated calcium channels, or the inversion of Na^+^/Ca^2+^ exchanger (Mandolesi et al., 2004). Secondly, the newly-formed growth cone always arises from the area where [Ca^2+^]_i_ is elevated (Ziv and Spira, 1997). Thirdly, a transient elevation of [Ca^2+^]_i_ to 300–500 μM in intact axons by microinjection of calcium ionophore leads to the formation of a new growth cone and neurite (Ziv and Spira, 1997). Fourthly, using electron microscopy, the region experiencing a [Ca^2+^]_i_ elevation has been observed to be also undergoing ultrastructural alterations and reorganisation of cytoskeletal structures, including microtubules and neurofilaments (Benbassat and Spira, 1993; Ziv and Spira, 1997). Nevertheless, it must be noted that the increase in [Ca^2+^]_i_ has to be a transient event. The ultrastructural change at the cut tip only occurs after the [Ca^2+^]_i_ has returned to its original level; and a new growth cone only begins to spread after the recovery of [Ca^2+^]_i_ (Benbassat and Spira, 1993; Ziv and Spira, 1997). After all, *Aplysia* axons completely re-seal after axotomy within 5 min, thus it makes sense that intracellular calcium buffers would be able to regulate the [Ca^2+^]_i_ thereafter (Gabso et al., 1997; Spira et al., 1993, 1996). The transient elevation of [Ca^2+^]_i_ then causes the activation of calcium-dependent proteases, which in turn carry out the process of protein degradation necessary for successful regeneration. Following the transection of an axon, intra-axonal proteolytic activity increases transiently, in a spatial and temporal distribution that closely resembles that of the transient [Ca^2+^]_i_ increase post-axotomy (Gitler and Spira, 1998). A similar rise in [Ca^2+^]_i_ is also thought to occur in mammalian axons. In cultured CNS neurons, axotomy leads to an [Ca^2+^]_i_ increase, which is thought to be the result of the opening of voltage-gated calcium channels by action potentials (Mandolesi et al., 2004). In addition, axotomy of DRG axons in a calcium-free medium greatly impairs growth cone regeneration (Chierzi et al., 2005).

Both the rise in proteolytic activity and the formation of the growth cone after axotomy can be inhibited by the application of calpeptin, an inhibitor of the calcium-dependent protease calpain, suggesting that calpain could be the mediator of the proteolysis (Gitler and Spira, 1998). In animal models of spinal cord injury, the level of calpain in spinal cord extracts from injured rats increases significantly within 1 h, compared to those from uninjured rats (Banik et al., 1997; Springer et al., 1997).

Several mechanisms have been proposed to explain the actual function of calcium/calpain-mediated proteolysis in axon regeneration after injury. One suggestion is that calpain helps in the initial re-sealing of the damaged plasma membrane after transection, as exhibited in invertebrate (Godell et al., 1997) and vertebrate models (Howard et al., 1999; Shi et al., 2000; Yoo et al., 2003). Other evidence points to the proteolysis of a submembrane cytoskeletal component, spectrin, that links the plasma membrane to intracellular cytoskeleton (Bennett and Baines, 2001). It has been suggested that the removal of spectrin facilitates the fusion of intracellular granules to the inner surface of the membrane during exocytosis (Aunis and Bader, 1988). Therefore, after axotomy, the upregulation of calpain-mediated breakdown of spectrin may lead to better fusion of axoplasmic vesicles to the membrane, helping the construction of a growth cone or extension of the axon (Gitler and Spira, 1998). Also, as mentioned above, axotomy of an *Aplysia* axon brings about a reorganisation of the cytoskeleton, involving an initial disassembly and breakdown of microtubules and neurofilaments, followed by the division of the cut axon tip into three zones (Sahly et al., 2006). If the axotomy is performed in the presence of calpeptin, these events fail to take place; but if calpeptin is added 4–5 min after axotomy, a growth cone with an expanding lamellipodium occurs, but at a much slower rate (Sahly et al., 2006). Besides, as alluded to before, sciatic nerve injury in rats triggers the synthesis of new vimentin that assists in the retrograde transport of signalling molecules such as pErk (see above). However, these newly synthesised vimentin molecules need to be broken down first by calpain-mediated proteolysis, and the resulting vimentin fragments are then able to transport pErk, along with importin β1 (Perlson et al., 2005; reviewed by Willis and Twiss, 2006).

*In vivo* experiments using rat spinal cord injury models have demonstrated that neurofilament, spectrin and microtubule-associated protein 2 (MAP2, albeit in the somatodendritic region) within the spinal cord undergo proteolysis induced by calpain (Banik et al., 1997; Springer et al., 1997). However, these studies appear to suggest that the proteolytic activity results in neuronal dysfunction, neuronal loss, tissue necrosis and even axon degeneration (Banik et al., 1997; Springer et al., 1997). Nevertheless, these seemingly contradicting results may be explained by the time-course of the proteolytic events studied, as well as the sample used. Firstly, experiments on cultured neurons looked at the rise of [Ca^2+^]_i_ within minutes, and a new growth cone normally re-forms within tens of minutes; whereas the increase in calpain activity in the *in vivo* studies occurred over 1 to 72 h (Banik et al., 1997; Spira et al., 2001; Springer et al., 1997). Indeed, the intra-axonal ultrastructural alterations that precede the formation of growth cone only takes place after the transient increase in [Ca^2+^]_i_ has ended (within minutes) and the [Ca^2+^]_i_ has returned to control level (Benbassat and Spira, 1993; Ziv and Spira, 1997). Secondly, the enhanced calpain activity in the whole spinal cord may have come from non-neuronal sources such as reactive glial cells and inflammatory cells, and not just limited to the tiny vicinity near the cut axon tip (Banik et al., 1997). It is possible that there are differences between mammalian and invertebrate axons. In a recent study of developing mouse hippocampal neurons application of the calpain inhibitor calpeptin to the axons of cultured hippocampal neurons induced neurite sprouting and a new growth cone (Mingorance-Le Meur and O'Connor, 2008). This discrepancy from the results from the Spira laboratory could reflect the use of different cell types, developmental stages or species. More work on mammalian models would provide more insights into understanding the actual role of calpain in growth cone formation. At present, most of the information about calcium changes in cut axons comes from *in vitro* experiments. Exactly what happens in mammalian PNS and CNS axons may differ somewhat from this picture, and there could be differences between central and peripheral axons.

### Possible role of autophagy in axon regeneration

Autophagy (self-eating) is the name used for any intracellular process that results in the degradation of cytoplasmic components inside lysosomes (Cuervo, 2004). It is thought to contribute toward cellular homeostasis by degrading long-lived stable proteins and appears to be the only mechanism by which entire organelles such as mitochondria, peroxisomes and regions of Golgi and ER are degraded. In addition, autophagy has also been implicated in cellular differentiation, growth control, cell death and neurodegenerative diseases (reviewed by Cuervo, 2004; Larsen and Sulzer, 2002; Rubinsztein et al., 2005). Components of the autophagic machinery have been detected in living neurons in culture and *in vivo* (Cubells et al., 1994; Hollenbeck, 1993; Rubinsztein et al., 2005). There are studies that suggest that axotomised neurons exhibit features resembling autophagic activity in the cell body and in the axon initial segment, including chromatolysis and the appearance of autophagosomes (Matthews, 1973; Matthews and Raisman, 1972; Rubinsztein et al., 2005). More recently, autophagy has been shown to be required for axonal homeostasis and prevention of degeneration in mouse Purkinje cells (Komatsu et al., 2007; Yue et al., 2008), but confusingly another report found that autophagy was induced in degenerating SCG neurites after nerve growth factor (NGF) withdrawal (Yang et al., 2007). These results suggest that autophagy might contribute toward the homeostasis and maintenance of the axons; future work is needed to elucidate its role in axons from other neuronal types and whether its activity might be linked to regeneration. The role of autophagy in axon regeneration remains speculative, as no direct evidence exists to associate autophagy-mediated degradation of proteins with the onset of the axonal regeneration programme.

## Conclusion

The first step to ensure successful regeneration after an axon is cut is the formation of a growth cone. However, a sequence of several other events must take place before the growth cone is formed. The axon will retract, pause, regenerate a growth cone and then elongate forward. This series of events can take place within one day *in vivo* (Friede and Bischhausen, 1980; Pan et al., 2003) and as quickly as a few hours *in vitro* (Chierzi et al., 2005; Verma et al., 2005). They also occur too rapidly for a cell body to receive retrograde signals formed at the injured site and to respond accordingly by sending the required proteins through axonal transport. The same holds true for components of the numerous proteolytic machineries that must be already in place to help degrade and depolymerise proteins during the retraction and subsequent phases (Campbell and Holt, 2001; Verma et al., 2005). Thus, the regeneration of an axon that culminates in the formation of a growth cone involves a complex and dynamic succession of molecular and anatomical events. An increasing number of reports are elucidating how local protein synthesis and degradation occur separately in the axon and growth cone in response to specific cues. However, it still remains unknown how the cellular machinery at the axon tip integrates and coordinates these processes in order to accomplish and maintain the appropriate levels of thousands of proteins necessary for regeneration. The future challenge is to understand how this complex network of processes interacts and cross-talks in order to promote regeneration after injury.
